# A Woman With Multiple Neuropathic Symptoms, Hypersensitivity to Heat, Peppers, and Alcohol, With a Variant in the TRPV1 Capsaicin Receptor Gene: Possible Implications to ME/CFS

**DOI:** 10.1155/crig/1920171

**Published:** 2026-11-03

**Authors:** Phillip M. Zimmermann, Richard G. Boles

**Affiliations:** ^1^ Mitochondrial & Molecular Medicine Pasadena California USA; ^2^ NeuroNeeds Old Lyme Connecticut USA

**Keywords:** alcohol intolerance, burning mouth, channelopathy, heat hypersensitivity, migraine, myalgic encephalomyelitis/chronic fatigue syndrome, transient receptor potential vanilloid 1, visceral hypersensitivity

## Abstract

Transient receptor potential vanilloid 1 (TRPV1) is a ligand and heat‐gated ion channel expressed on nociceptors that plays a central role in thermal and chemical pain signaling. Loss‐of‐function variants in TRPV1 have been associated with reduced nociception. We report a 65‐year‐old woman with disabling polyneurofunctional/neuropathic symptomatology including migraine, functional gastrointestinal disorders, and myalgic encephalomyelitis/chronic fatigue syndrome (ME/CFS). Diagnostic evaluation revealed sensory polyneuropathy and autonomic instability. Whole genome sequencing reanalysis identified a heterozygous TRPV1 c.2174A > T (p.Lys725Met) Likely Pathogenic variant that is very rare and highly conserved. Our patient reports consistent, longstanding, marked hypersensitivity to heat, capsaicin, and alcohol, all of which are known TRPV1 agonists, supporting a gain‐of‐function mechanism for this variant. Furthermore, prior studies have implicated TRPV1 hyperactivity in migraine, nociceptive sensitization, and ME/CFS. Despite the excellent clinical correlation, causality of the variant cannot be definitively established without functional studies. This case expands the potential phenotypic spectrum associated with TRPV1, and highlights a possible role for TRPV1‐mediated channelopathies in complex neurofunctional disorders including ME/CFS.

## 1. Introduction

The human transient receptor potential vanilloid 1 (TRPV1) gene encodes a ligand and heat‐gated, calcium‐permeant, nonselective cation channel that is expressed on nociceptors [1]. The channel is activated by heat > 42°C and extracellular protons (acid). Additionally, the TRPV1 channel is activated by the vanilloid capsaicin, which is the active ingredient that makes chili peppers feel “hot” and thus this channel is often referred to as the capsaicin receptor. Furthermore, TRPV1 can also be activated by endogenous lipid mediators, including anandamide and N‐arachidonoyl dopamine [2]. The channel is believed to be a key mediator of thermal and chemical pain, and TRPV1 channel signaling is believed to play important roles in sensory hyperalgesia [1, 3].

We report a 65‐year‐old woman with marked heat, capsaicin, and alcohol hypersensitivity with multiple clinical manifestations of neuropathy and myalgic encephalomyelitis/chronic fatigue syndrome (ME/CFS). Reanalysis of singleton whole genome sequencing (WGS) identified a heterozygous TRPV1 c.2174A > T (p.Lys725Met) variant. These observations support this as a gain‐of‐function variant that amplifies TRPV1 channel function and may have potential implications to the pathophysiology of ME/CFS.

## 2. Case Description

The patient is a 65‐year‐old Caucasian female medical professor who was healthy until age 57. Three days after receiving an influenza vaccination, she developed new‐onset orthostatic intolerance without other identified triggers. About 5 weeks later, she experienced a significant deterioration in her health with the onset of severe malaise, nausea, dizziness, disequilibrium, fatigue, and brain fog. Overtime, she came to meet diagnostic criteria for ME/CFS, including severe life‐altering fatigue, postexertional malaise, nonrestorative sleep, orthostatic changes, and cognitive dysfunction [4]. These symptoms forced her into early retirement.

At the time of our evaluation, her chief complaints were persistent severe fatigue and multiple neuropathic‐like symptoms with chronic migraine with aura, urinary frequency, tenesmus, dizziness, nausea, paresthesia, areas of anesthesia, loss of proprioception, muscle stiffness, tinnitus, and visual hypersensitivity. Hot showers, heating pads, direct sunlight, and alcohol intake also trigger flares of her chief symptoms. In contrast, she prefers cold weather. Issues that predated her rapid descent into illness and disability at age 57 included substantial and frequent, spontaneous burning mouth pain that she describes as feeling like she is “eating hot peppers.” She has had longstanding intolerance to even minimally spicy foods. A pepper dip in her mid 40s caused laryngospasm. Family history is only notable for her mother, who developed rapidly progressive “multiple sclerosis” at age 52 and died at age 53.

Electromyography revealed a mild sensory polyneuropathy. Tilt table testing induced asystole within 35 s, requiring cardiopulmonary resuscitation. A pacemaker was implanted but has never been activated. A left vastus lateralis muscle biopsy showed mild, nonspecific, neurogenic changes, including mild type II fiber hypotrophy. Electron microscopy revealed mitochondrial subsarcolemmal aggregates. Mitochondrial respiratory chain enzymology from muscle showed partial deficiencies of complex II (41.4, 5^th^–95^th^ %: 49.0–133.4) and citrate synthase (119.1, 5^th^–95^th^ %: 159.8–353.3). The biopsy site remained warm for several months after the procedure.

Singleton WGS was performed at a commercial laboratory (Variantyx, Framingham, MA). The official clinical report was negative. However, comprehensive reanalysis of the sequence identified a heterozygous TRPV1 c.2174A > T (p.Lys725Met; chr17:3,572,179) variant. This variant is extremely rare in the population (reported once in roughly 800,000 people at the time of the review [5]). The lysine at position 725 is highly conserved across vertebrate species, suggesting functional importance. Multiple computer‐based tools predicted this variant to alter protein function, with a very‐high Aggregate Predicted Severity Score per Variantyx software of 0.8. With the addition of protein functional domain criteria, this variant would be annotated as “Likely Pathogenic” per ACMG criteria [6].

## 3. Discussion

Individuals with loss‐of‐function variants in TRPV1 exhibit marked reductions to heat‐related pain and capsaicin [2]. Consistent with this, TRPV1‐deficient mice reveal reduced responses to heat, protons, and vanilloid agonists including capsaicin [7].

In contrast, the clinical picture in our case strongly suggests heightened responsiveness (gain‐of‐function) of the TRPV1 system. The heterozygous p.Lys725Met variant identified in our case is located in the proximal C‐terminal regulatory region of the TRPV1 protein. This domain is essential for channel gating, trafficking, and sensitization, and variants therein can shift activation thresholds for channel excitability [8]. In our case, heat, capsaicin, and alcohol exposure all reliably trigger or worsened her symptoms. TRPV1 is well known to be activated by heat and capsaicin. Even low concentrations of alcohol significantly potentiate TRPV1 activation and lower its heat activation threshold, producing burning sensations [9]. Thus, our case’s burning mouth pain is consistent with overactivation of TRPV1‐expressing nociceptors, which are present in oral mucosa [10]. In addition to TRPV1, TRPA1 and TRPM3 contribute to thermal sensation [11]. However, a gain‐of‐function variant TRPV1, or perhaps any one of those genes, could be of such magnitude to result in highly increased heat sensation via dominant inheritance.

Our case’s migraine with aura is potentially compatible with TRPV1 involvement in trigeminal neuropeptide release resulting from heightened trigeminal pathway sensitivity [3]. As afferent signaling to bladder distention was “significantly attenuated” in TRPV1 knockout mice, urinary frequency in our case likely is consistent with TRPV1 hyperactivity [12]. In studying rectal biopsy specimens from people with irritable bowel syndrome compared to healthy controls, the authors in [13] reported that TRPV1 agonists resulted in heightened visceral pain, urgency, inflammatory flare responses, and colorectal hypersensitivity, yet only in the IBS group. Thus, functional gastrointestinal symptoms such as tenesmus in our case could be explained by oversensitized TRPV1 expressing fibers in the colorectum.

ME/CFS is a relatively common and oftentimes highly disabling condition that remains essentially idiopathic. Symptoms become markedly worse following exercise. In a rat model of ME/CFS, both a TRPV1 agonist (capsaicin) and an antagonist (n‐tert‐butylcyclohexanol) reversed the increase in immobility period induced by forced swimming. This effect was “attributed to inhibition of (the) TRPV1 channel” [14]. In humans with ME/CFS, TRPV1 expression “demonstrated a consistent although nonsignificant trend” to be elevated at all time points postexercise, which was not observed in healthy controls [15]. These studies suggest that overactivity of TRPV1 might contribute toward ME/CFS and may suggest a potential path for therapy; however, this remains speculative and requires additional study. On the other hand, the TRPV1 variant identified may be unrelated to the development of ME/CFS, or perhaps other phenotypes, in our case.

Our case describes a distinct multisystem sensory and autonomic phenotype that is consistent with heightened TRPV1 responsiveness, presumably resulting from a Likely Pathogenic missense variant in TRPV1. This case expands the recognized spectrum of TRPV1‐associated disease and emphasizes the importance in considering TRPV1, and abnormalities of ion channels (channelopathy) in general, as a potential factor in cases with varied neurofunctional disorders such as migraine, interstitial cystitis, functional gastrointestinal conditions, and/or ME/CFS. A significant limitation is the lack of functional studies to verify and understand the pathogenicity of the genetic variant.

## Author Contributions

Conceptualization: Richard G. Boles.

Methodology: Phillip M. Zimmermann and Richard G. Boles.

Investigation: Phillip M. Zimmermann and Richard G. Boles.

Resources: Richard G. Boles.

Data curation: Phillip M. Zimmermann and Richard G. Boles.

Writing–original draft: Phillip M. Zimmermann and Richard G. Boles.

Writing–review and editing: Phillip M. Zimmermann and Richard G. Boles.

Supervision: Richard G. Boles.

Project administration: Richard G. Boles.

## Funding

No funding was received for this research.

## Consent

Written informed consent was obtained from the patient.

## Conflicts of Interest

The authors declare no conflicts of interest.

## Data Availability

The data that support the findings of this study are available on request from the corresponding author. The data are not publicly available due to privacy or ethical restrictions.
